# Supplementary material for: Atomically precise engineering of spin–orbit polarons in a kagome magnetic Weyl semimetal
Source: Nat Commun. 2024 Mar 14;15:2301. doi: 10.1038/s41467-024-46729-3 (PMC10940584; doi:10.1038/s41467-024-46729-3)
Supplement: Supplementary file 1 — Supplementary information [file 41467_2024_46729_MOESM1_ESM.pdf]

Supplementary Information for

Atomically precise engineering of spin–orbit polarons in a kagome magnetic Weyl semimetal

Hui Chen[1,2,3]†, Yuqing Xing[1,2]†, Hengxin Tan[4], Li Huang[1,2], Qi Zheng[1,2], Zihao Huang[1,2], Xianghe Han[1,2], Bin Hu[1,2], Yuhan Ye[1,2], Yan Li[1,2], Yao Xiao[1,2], Hechang Lei[5], Xianggang Qiu[1], Enke Liu[1], Haitao Yang[1,2,3], Ziqiang Wang[6], Binghai Yan[4] and Hong-Jun Gao[1,2,3*]

Corresponding author: hjgao@iphy.ac.cn,

† These authors contributed equally to this work

Section I. Additional AFM and STM/S characterization of vacancy repair
Supplementary Figure 1. Demonstration of the single vacancy with nc-AFM.
Supplementary Figure 2. Typical large-scale STM image of S-terminated surface.
Supplementary Figure 3. Demonstration of the single vacancy repairing with nc-AFM.

Section II. Additional data of STS and calculations to support the vacancy repair mechanism
Supplementary Figure 4. Topography and d*I*/d*V* spectra of S vacancies before and after vacancy repair.
Supplementary Figure 5. Transition barrier of a sulfur atom from the bulk to the single vacancy site on the S-surface.
Supplementary Figure 6. Comparison between artificially-created vacancy and naturally-formed vacancies.

Section III. Additional data of step-by-step atomic manipulation
Supplementary Figure 7. Step-by-step atomic manipulation of S vacancy aggregates

Section IV. Additional STS data of artificially-created vacancies with designed shape
Supplementary Figure 8. Two spatially-separated single vacancies with decreasing spacing.
Supplementary Figure 9. Localized quantum states around a dimer S-vacancy at the S-terminated surface.
Supplementary Figure 10. STM images and spectra of five-, six-, and seven- vacancies on S terminated surfaces.
Supplementary Figure 11. Comparison between upward and downward triangular vacancies.
Supplementary Figure 12. d*I*/d*V* linecut along the edge of linear atomic S vacancies.
Supplementary Figure 13. d*I*/d*V* maps of large-size triangular S vacancies.

Section V. Calculated energy of bound state with vacancy size and shape
Supplementary Figure 14. Energy evolution of bound states for different vacancies.

Section VI. More information of strain analysis and peak fitting
Supplementary Figure 15. Analysis of the lattice distortion around S vacancies.
Supplementary Figure 16. Gaussian fitting of the d*I*/d*V* spectra in Fig. 2d and Fig. 4d.

Section VII. Extension of vacancy manipulation
Supplementary Figure 17. Vacancy manipulation in a $Co_3Sn_2S_2$-derived kagome metal, $Ni_3In_2S_2$.
Supplementary Figure 18. Engineering of the S atoms inside large-size vacancy.

Section VIII. Comparison between STS and magnetization measurements
Supplementary Figure 19. B_z dependent d*I*/d*V* spectra around S vacancies and magnetization of $Co_3Sn_2S_2$ crystal.

Supplementary Figure 1.

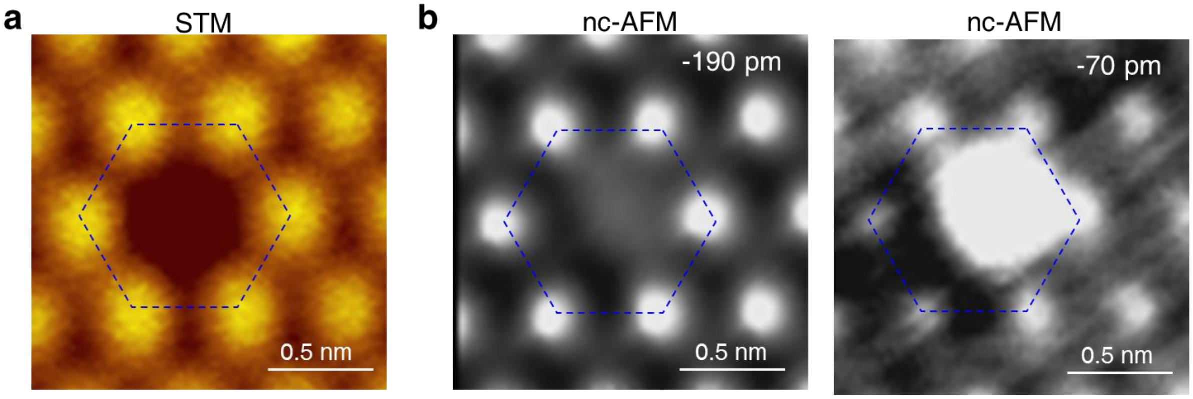

Supplementary Figure 1. Demonstration of the single vacancy with noncontact atomic force microscope (nc-AFM). a, STM image of the single S vacancy, showing a hole-like feature (V_s= -4 mV, I_t=10 pA). **b**, Constant-height AFM images by using a CO tip in the same region of (**a**) with the scanning height of -190 pm (left) and -70 pm (right), respectively, showing the missing S atom at the vacancy site. Scanning height: -190 pm, V_s= -4 mV, I_t=10 pA. Scanning amplitude = 50 pm.

Supplementary Figure 2.

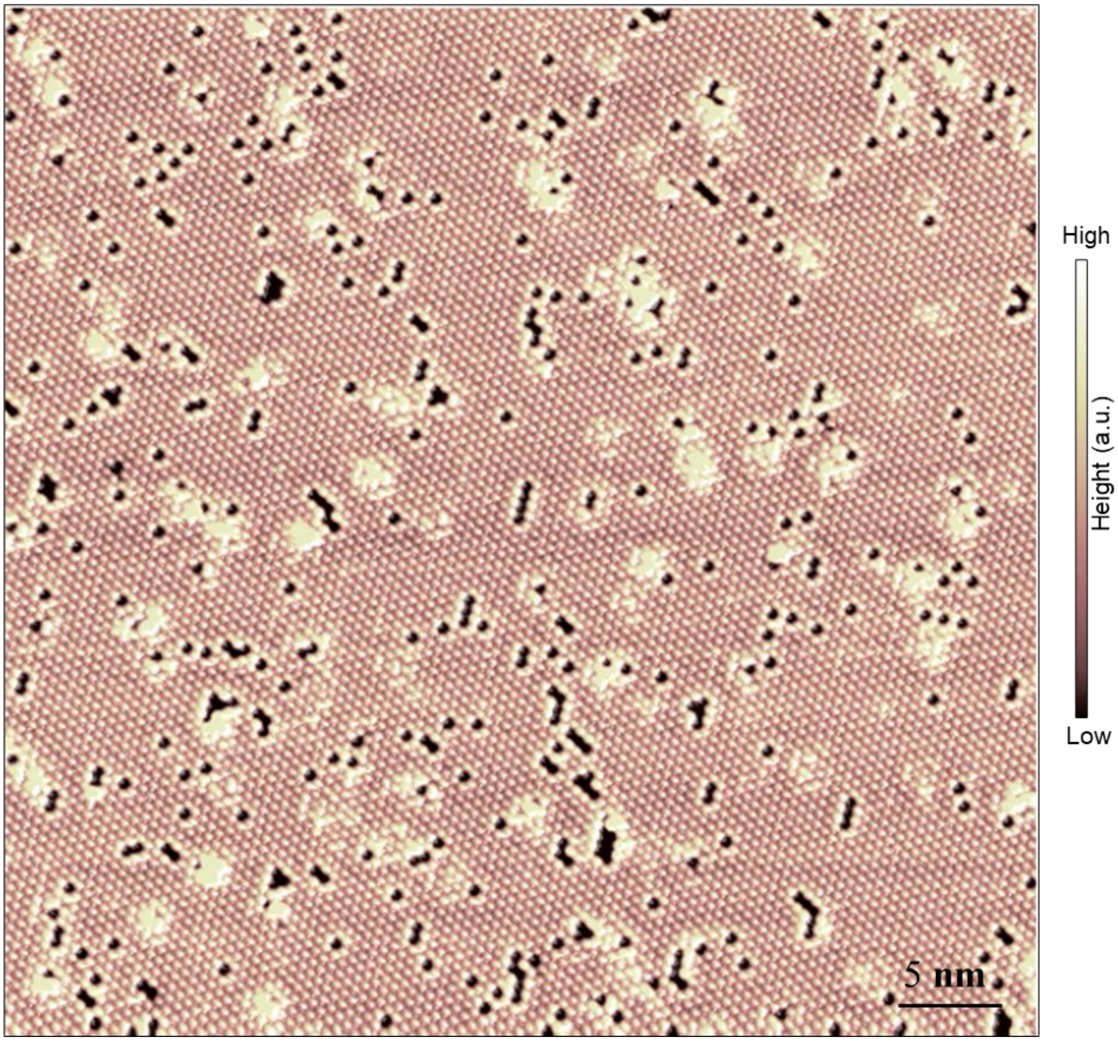

Supplementary Figure 2. Typical large-scale STM image of S-terminated surface, showing randomly-distributed S vacancies with different size and shape. STM parameters: V_s = −600 mV, I_t = 100 pA.

Supplementary Figure 3.

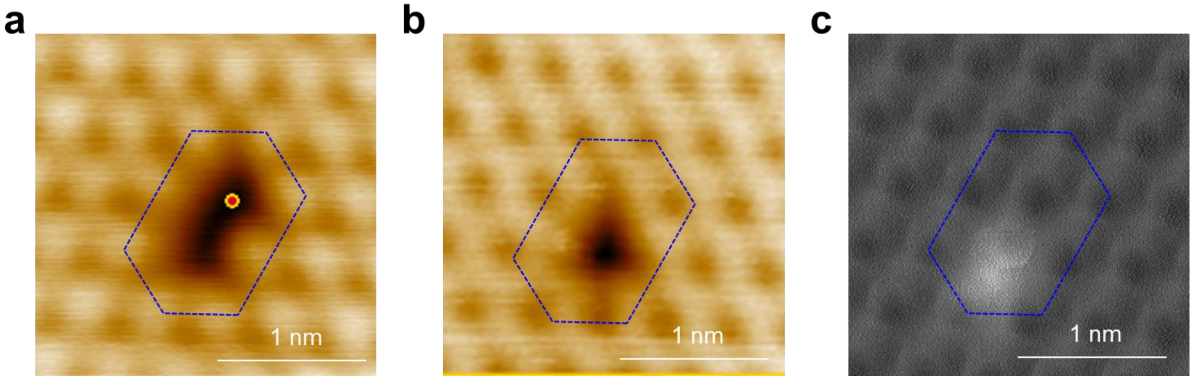

Supplementary Figure 3. Demonstration of the single vacancy repairing with nc-AFM. a,b STM images showing the topography before (**a**) and after (**b**) vacancy repairing (V_s= -4 mV, I_t=10 pA). **c,** Constant-height AFM images at the same region of (b), showing the filling of an additional S atom at the vacancy site as marked by the red spot in (**a**). Scanning height: -190 pm, V_s= -4 mV, I_t=10 pA. Scanning amplitude = 50 pm.

Supplementary Figure 4.

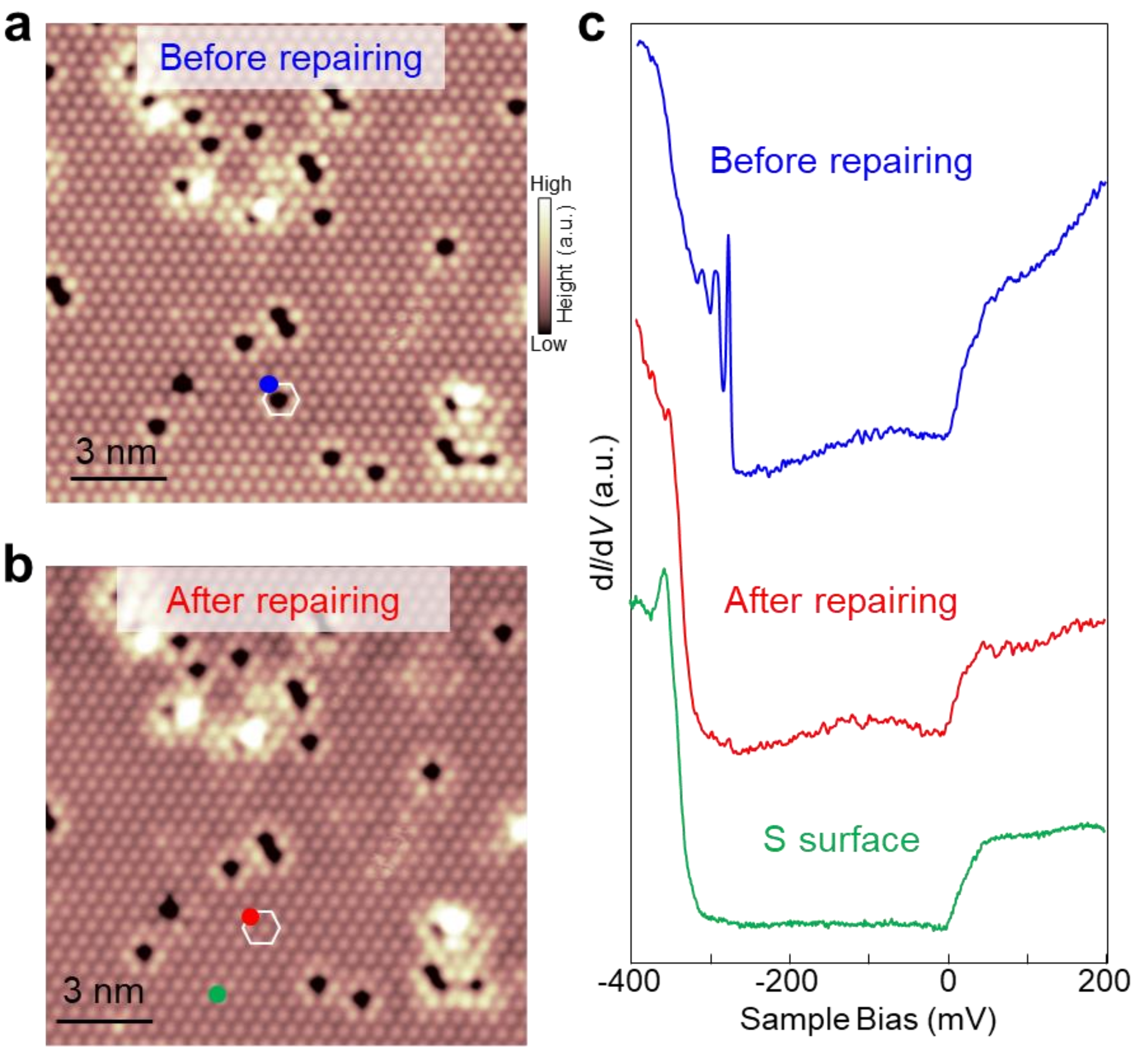

Supplementary Figure 4. Topography and d*I*/d*V* spectra of S vacancies before and after vacancy repair. a,b STM images before (**a**) and after (**b**) STM manipulations, showing that a single S vacancy is repaired ($V_s = -400$ mV, $I_t = 500$ pA). **c,** Typical d*I*/d*V* spectra obtained at the blue, red and green spots, respectively, showing the similar density of state at the as-repaired vacancy with vacancy-free S surface region ($V_s = -400$ mV, $I_t = 500$ pA, $V_{mod} = 0.5$ mV).

Supplementary Figure 5.

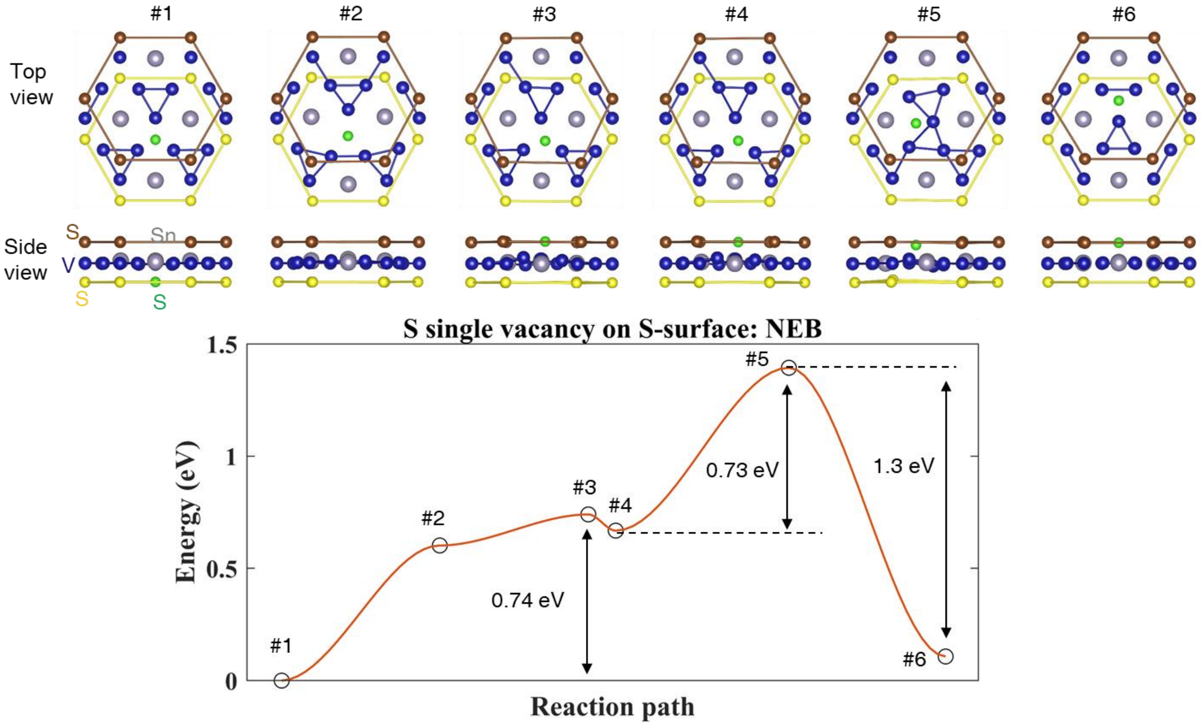

Supplementary Figure 5. Transition barrier of a S atom from the bulk to the single vacancy site on the S-surface. Four intermediate structures in the Nudged Elastic Band (NEB) calculations are considered as #2 ~#5, where #1 and #6 show the surface S vacancy and bulk S vacancy respectively (only the three top-most layers are shown for simplicity). The S (green) moves in out-of-plane direction from #1 to #3 (barrier ~ 0.74 eV), and moves in the surface plane from #3 to #6 (barrier ~ 0.73 eV). Notice that structures #3 and #4 are very close but not the same. The medium energy barrier indicates that pulling one S from the bulk to occupy the surface vacancy site is possible. Spin–orbit coupling is not included.

Supplementary Figure 6.

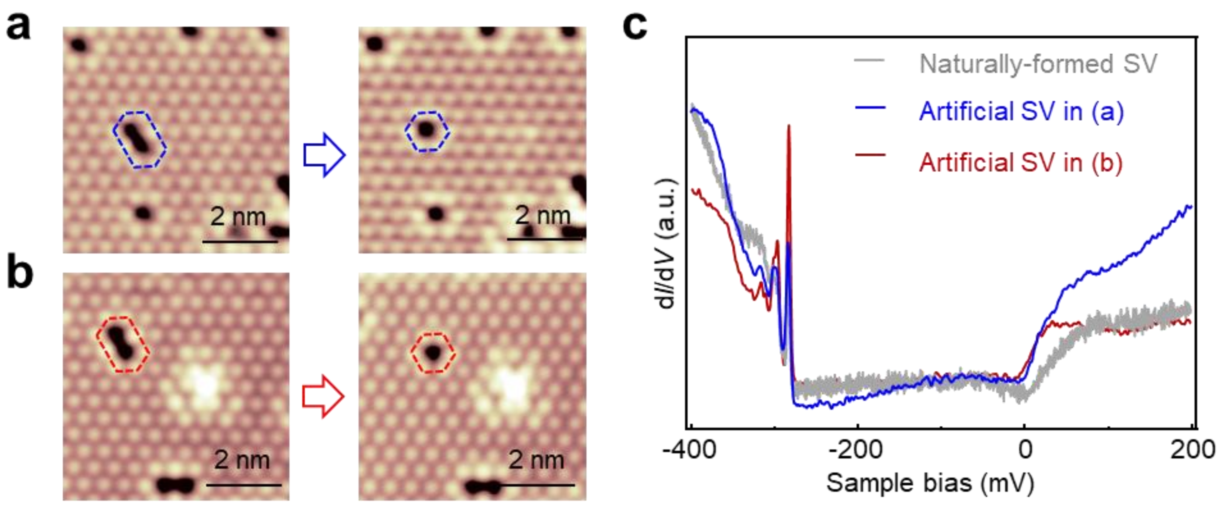

Supplementary Figure 6. Comparison of bound states between various artificially created vacancy and naturally formed vacancies. a,b STM images showing two cases of repairing a dimer vacancy into a single vacancy. (V_s = −400 mV, I_t = 500 pA). **c,** The dI/dV spectra obtained at artificial single vacancies in (**a**) and (**b**), showing similar features with the naturally formed single vacancies near Fermi level. (V_s = −400 mV, I_t = 500 pA, V_{mod} = 0.5 mV).

Supplementary Figure 7.

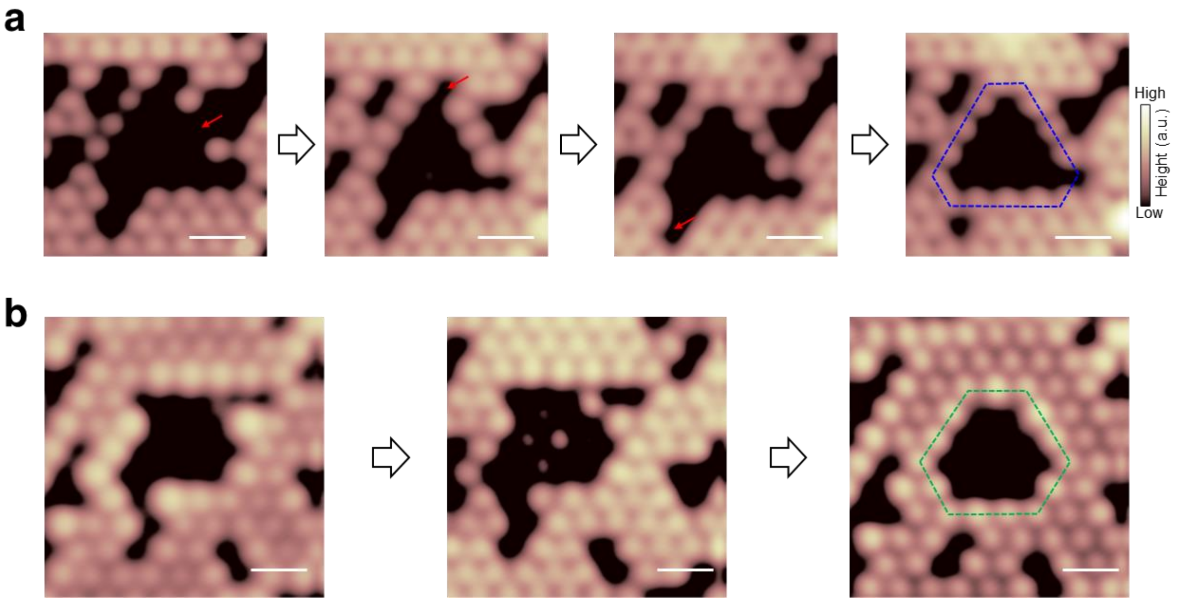

Supplementary Figure 7. Step-by-step atomic manipulation of S vacancy aggregates: from irregular to regular vacancy shape. a, Consecutive STM images of a large vacancy being repaired and transformed into a quasi-triangular vacancy highlighted by the dotted blue triangle ($V_s = -100$ mV, $I_t = 500$ pA). **b,** Consecutive STM images of another large vacancy being repaired and transformed into a quasi-hexagonal vacancy highlighted by the dotted green hexagon ($V_s = -100$ mV, $I_t = 500$ pA). The scale bars in all figures correspond to 1 nm.

Supplementary Figure 8.

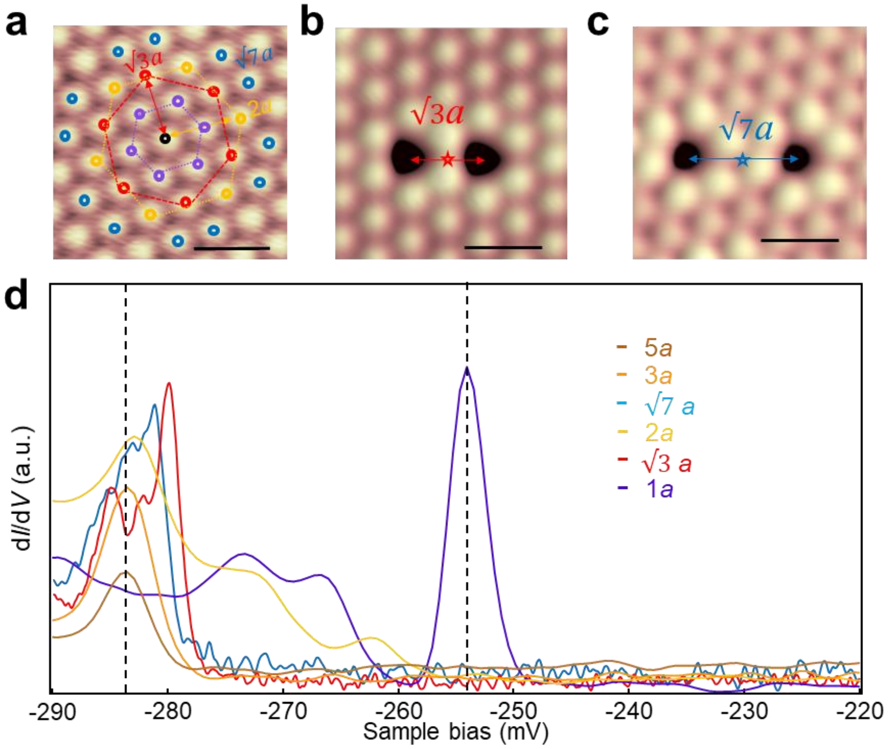

Supplementary Figure 8. Two spatially separated single vacancies with decreasing spacing. **a,** STM image of perfect S surface showing the nearest-neighbor (purple), second-nearest-neighbor (red), third-nearest-neighbor (yellow) and forth-nearest-neighbor atoms (cyan) around the center S atom. ($V_s = -400$ mV, $I_t = 500$ pA). **b,c** STM images of two spatially-separated vacancy with second-nearest-neighbor and forth-nearest-neighbor distance. **d**, dI/dV spectra for two spatially-separated vacancy with decreasing spacing L, showing that the primary peak shifts when L<3a (a is the lattice constant of $Co_3Sn_2S_2$) ($V_s = -300$ mV, $I_t = 500$ pA, $V_{mod} = 0.5$ mV). The scale bars in all figures correspond to 1 nm.

Supplementary Figure 9.

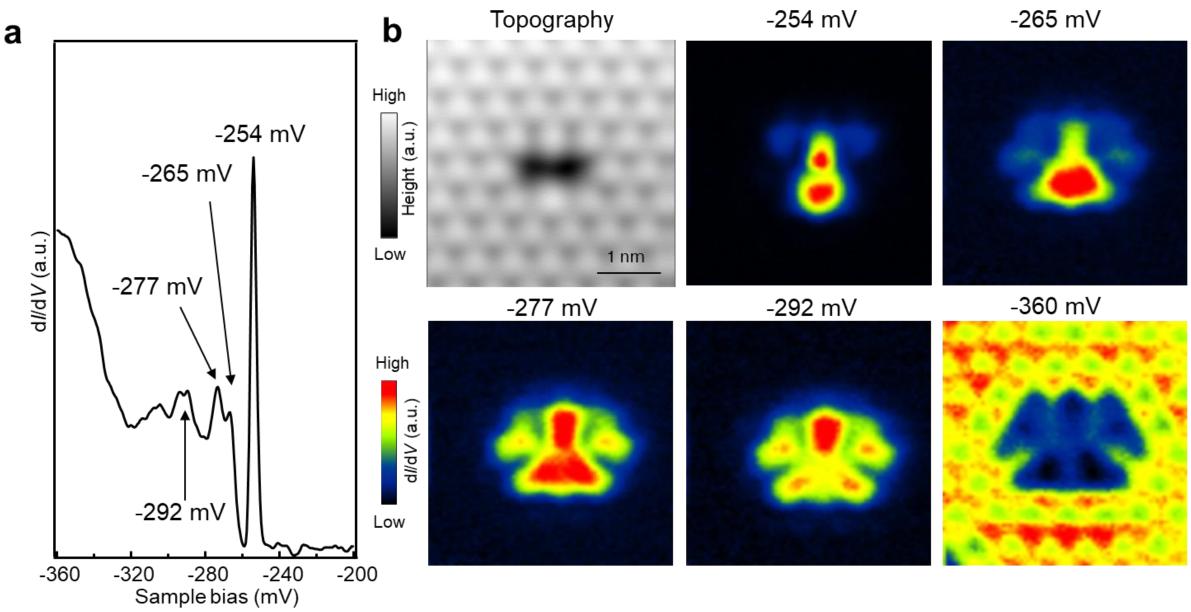

Supplementary Figure 9. Localized quantum states around a dimer S vacancy at the S-terminated surface. a, d*I*/d*V* spectra at a dimer vacancy, showing several peaks corresponding to the bound states induced by the vacancy. **b,** STM image of a dimer vacancy and d*I*/d*V* maps at different energies: -254 meV, -265 meV, -277 meV, -292 meV, and -360 meV, respectively. V_s = −360 mV, I_t = 500 pA, V_{mod} = 0.2 mV

Supplementary Figure 10.

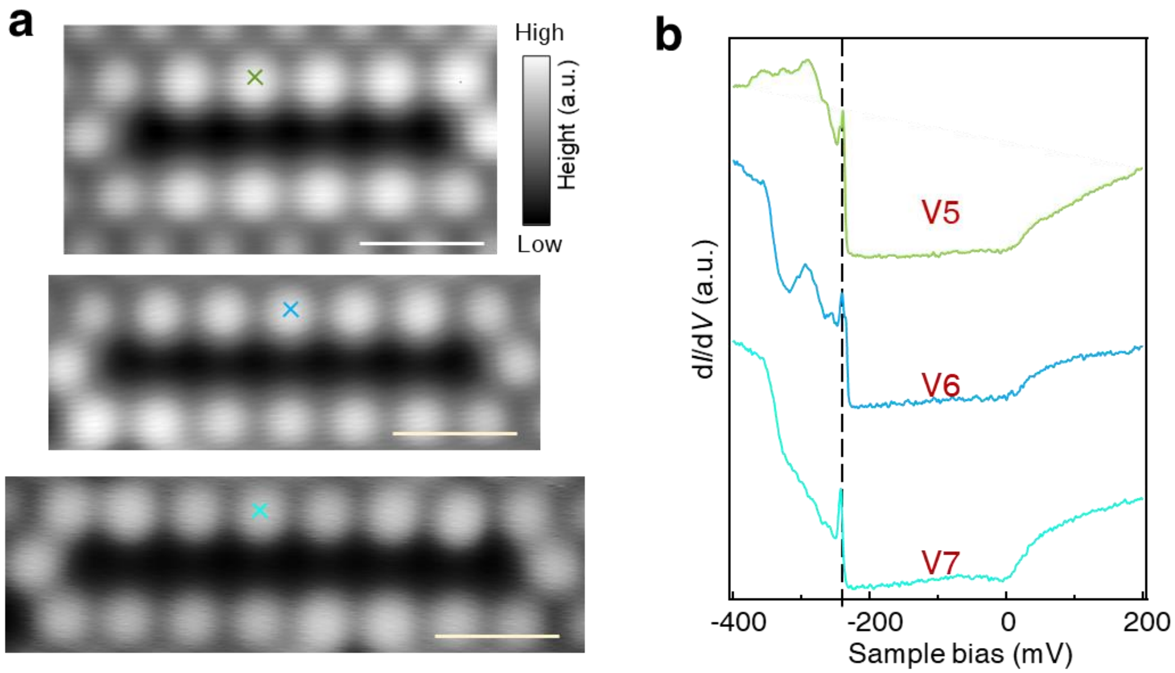

Supplementary Figure 10. STM images and spectra of five-, six-, and seven- atomic vacancies on S terminated surfaces. a, STM images showing five-, six-, and seven- atomic vacancies on S-terminated surfaces. **b,** dI/dV spectra on the positions (marked by light blue, blue and green cross in (**a**), respectively) of the five-, six-, and seven- atomic vacancies, respectively, showing the sharp peak around -240 mV. The scale bar is 1 nm in (**a**) and (**b**). $V_s = -400$ mV, $I_t =$ 500 pA, $V_{mod} = 0.2$ mV.

Supplementary Figure 11.

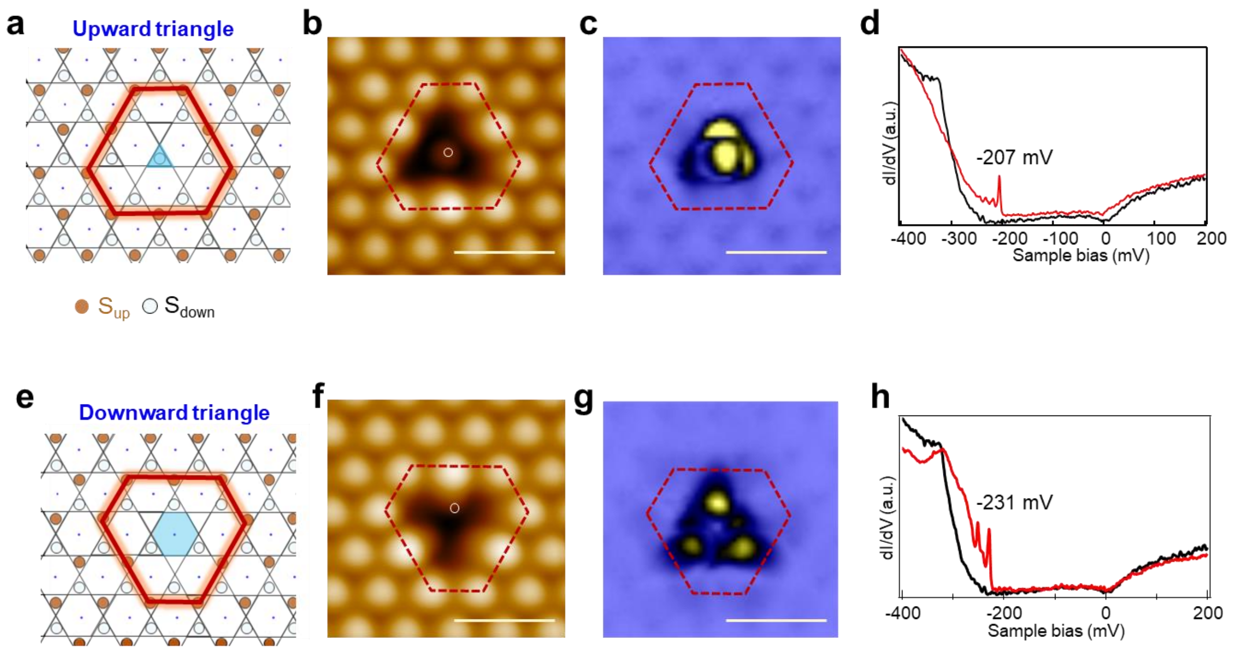

Supplementary Figure 11. Comparison between upward and downward triangular vacancies. **a**, Schematic showing the upward triangular vacancy where a Co-based triangle of the Co_3Sn kagome unit is centered in vacancy. **b-d**, STM image, d*I*/d*V* map at -207 mV and d*I*/d*V* curves obtained at the position marked by while circle in (**b**), respectively, showing the primary bound state is strongest at the vacancy center. **e**, Schematic showing the downward triangular vacancy where a hexagon of Co_3Sn kagome unit is centered in vacancy. **f-h**, STM image, d*I*/d*V* map at -231 mV and d*I*/d*V* curves obtained at the position marked by while circle in (**f**), respectively, showing the distribution of primary bound state. The scale bars in all figures correspond to 1 nm. STM parameters: $V_s = -400$ mV, $I_t = 500$ pA, $V_{mod} = 0.2$ mV.

Supplementary Figure 12.

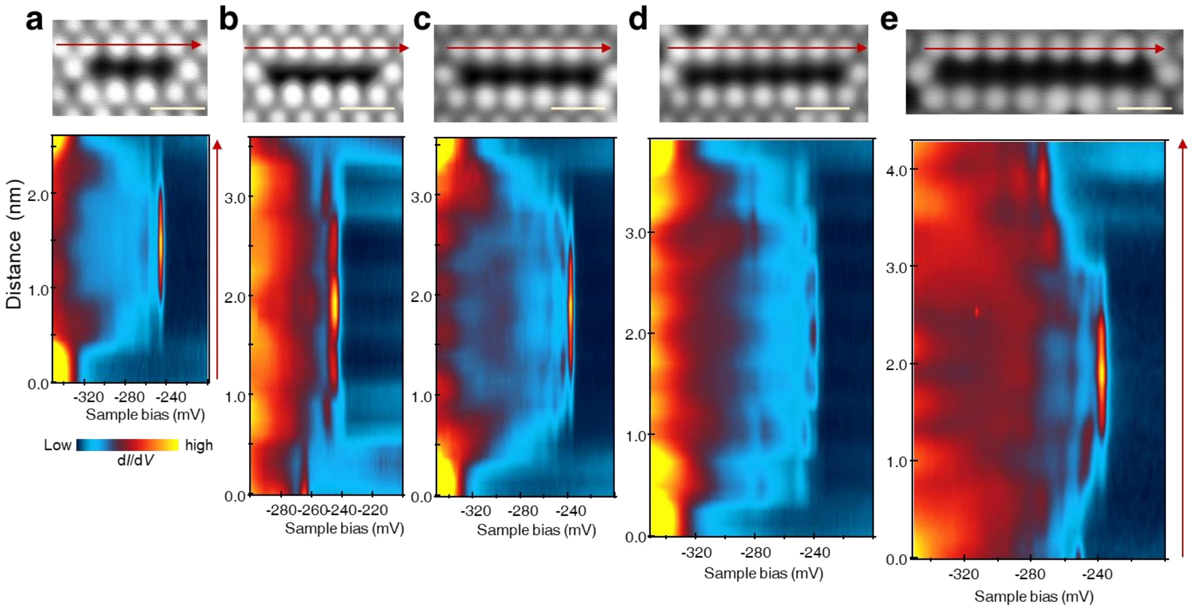

Supplementary Figure 12. The d*I*/d*V* linecut along the edge of linear atomic S vacancies. a-e STM topography of linear atomic vacancies and d*I*/d*V* linecut along the red arrow, showing the spatial decay of the bound states with the atomic shift. The scale bar is 1 nm. $V_s = -300$ mV, I_t = 500 pA, V_{mod} = 0.5 mV.

Supplementary Figure 13.

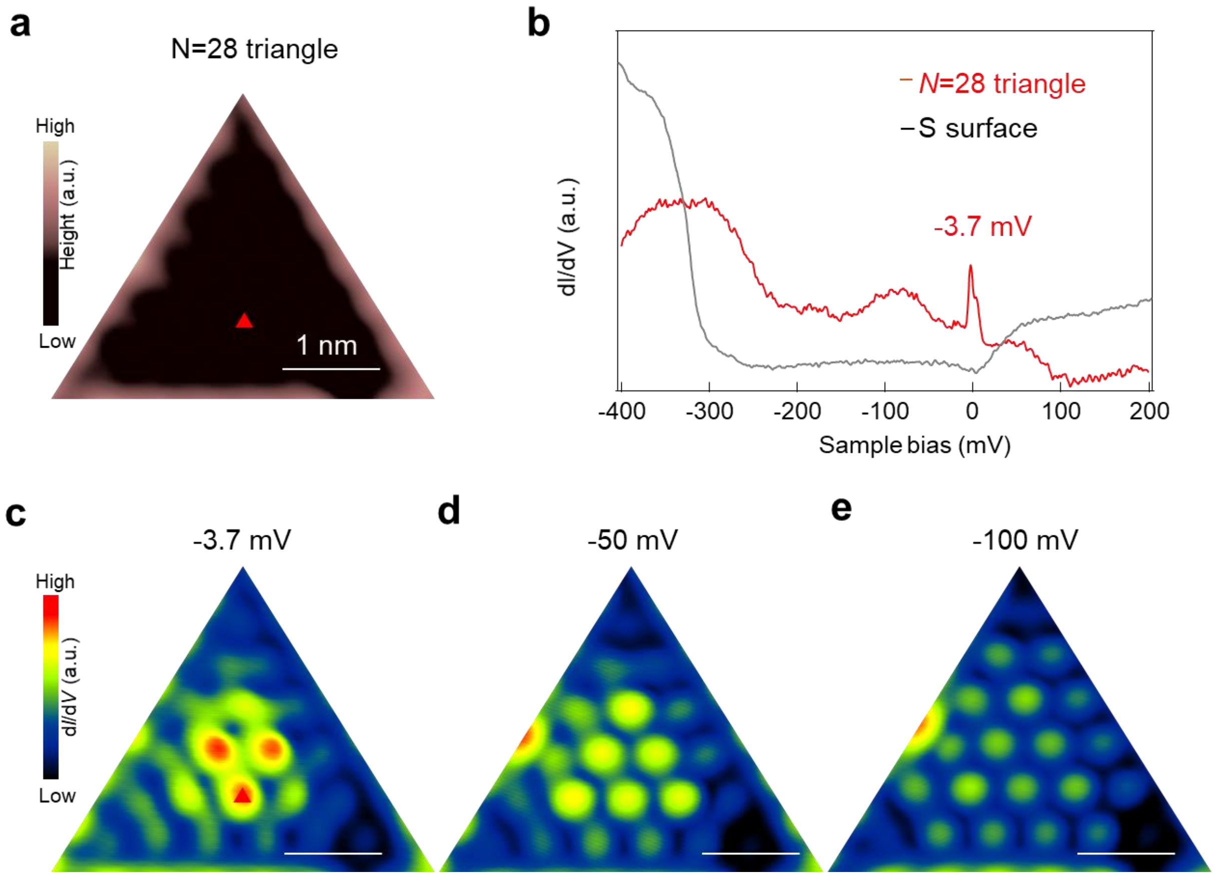

Supplementary Figure 13. d*I*/d*V* maps of large-size triangular S vacancies. a, STM topography of large-size triangular vacancy (*N*=28). **b**, d*I*/d*V* spectra obtained at the vacancy center (red triangle in (**a**)) and the S surface, respectively, showing the sharp conductance peak at -3.7 mV. **c**-**e**, the d*I*/d*V* maps at -3.7 mV (**c**), -50 mV (**d**) and -100 mV (**e**), respectively, showing the quantum confinement. STM parameter: $V_s = -400$ mV, $I_t = 500$ pA, $V_{mod} = 0.5$ mV.

Supplementary Figure 14.

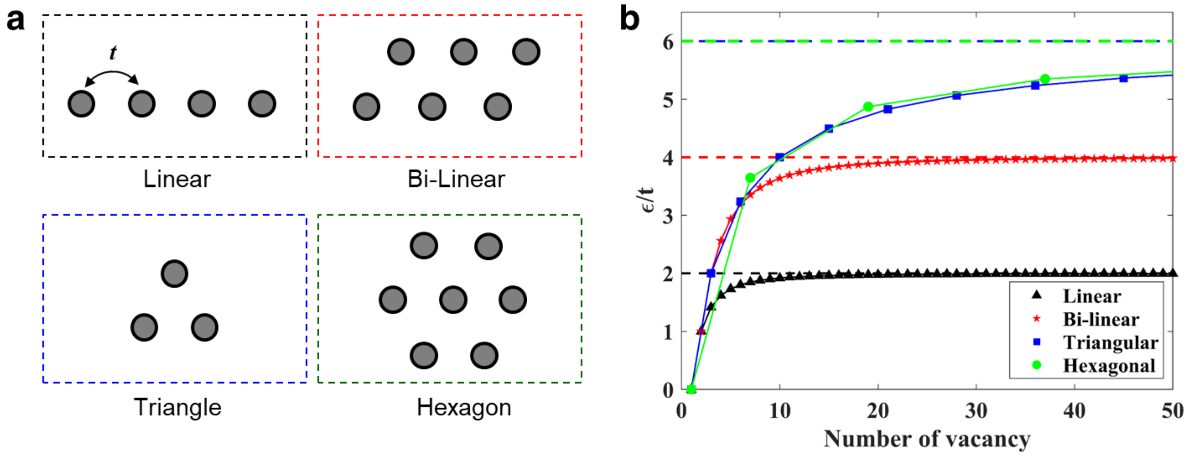

Supplementary Figure 14. Energy evolution of anti-bonding states for different vacancies. a, The model with a single hopping parameter, *t*, is considered in for simulation of the inter-vacancy interaction. The linear, bilayer and triangular (also hexagonal) configurations are considered. **b**, Energy evolution of the anti-bonding states for different vacancies based on the model in (**a**). In the vacancy number, the relative energy levels (ε/t) saturate to corresponding infinite size cases, $2t$, $4t$ and $6t$ for linear, bilayer and triangular (also hexagonal) configurations.

Supplementary Figure 15.

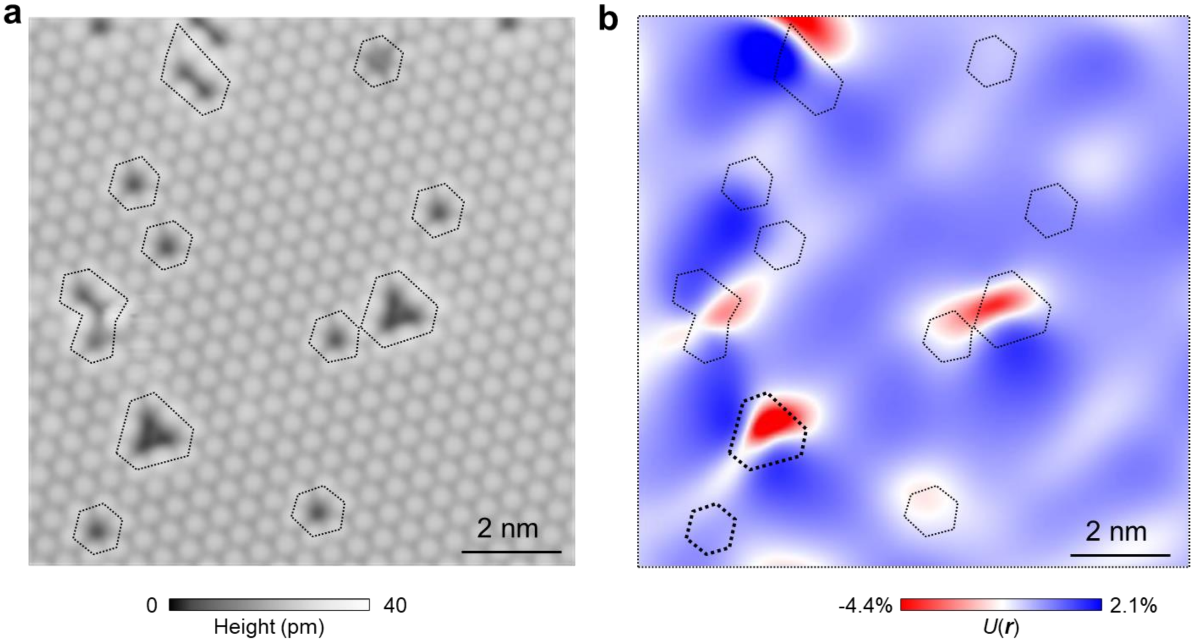

Supplementary Figure 15. Analysis of the lattice distortion around S vacancies. The antisymmetric strain map $U(\boldsymbol{r}) \equiv u_{aa}(\boldsymbol{r})\text{-}u_{bb}(\boldsymbol{r})$(**a**) are derived from STM topography (**b**), showing that the relatively-larger lattice distortion is localized at the triangular vacancies. $V_s = -400$ mV, $I_t = 500$ pA。

Supplementary Figure 16.

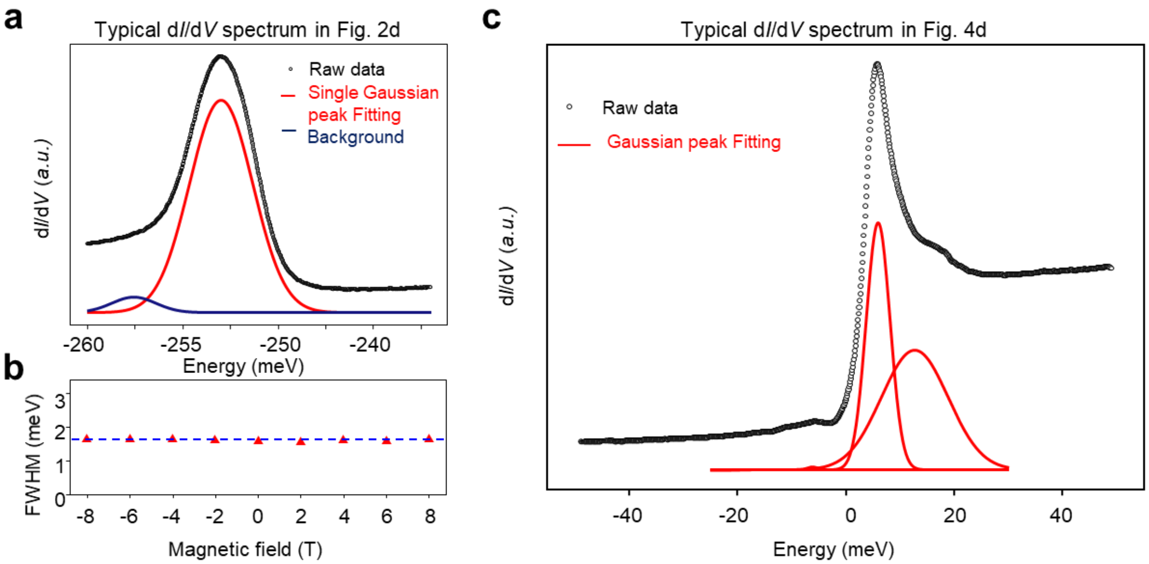

Supplementary Figure 16. Gaussian fitting of the d*I*/d*V* spectra in Fig. 2d and Fig. 4d. a, Fitting of one typical d*I*/d*V* spectrum in Fig. 2d, showing that the peak can be fitted by a simple Gaussian peak. **b**, Evolution of FWHM (full width at half maximum) subtracted from Gaussian fitting with magnetic field, showing no significant variation with the field. **c**, Fitting of one typical d*I*/d*V* spectrum in Fig. 2d, showing that the peak cannot be fitted by a simple Gaussian peak.

Supplementary Figure 17.

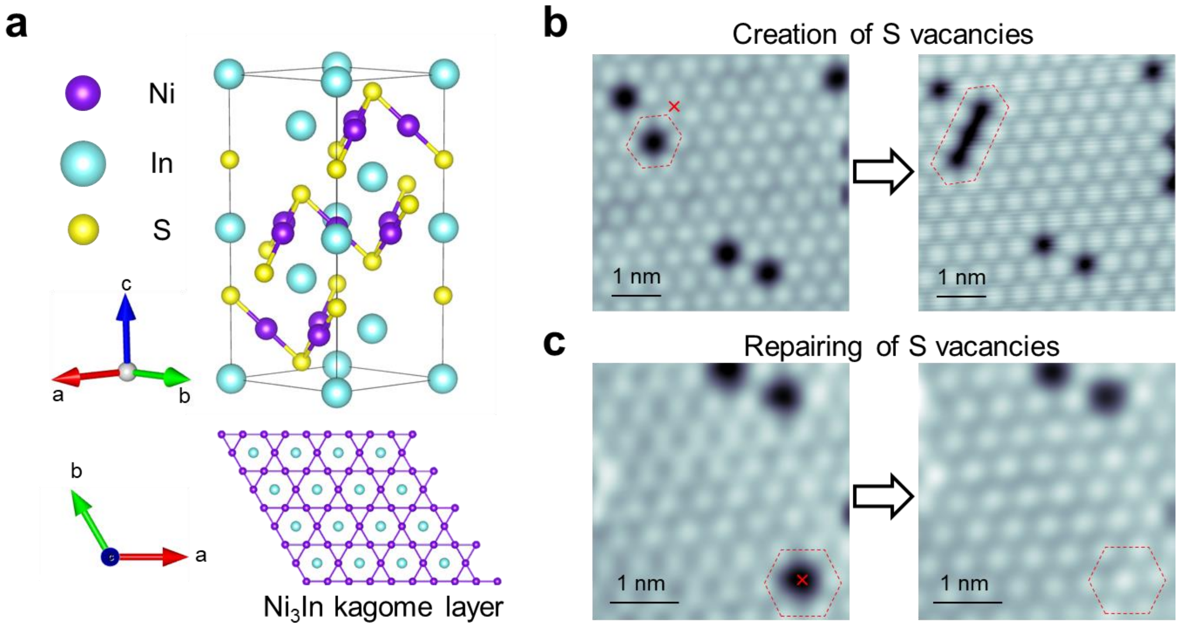

Supplementary Figure 17. Vacancy manipulation in a $Co_3Sn_2S_2$ derived kagome metal, $Ni_3In_2S_2$. **a**, Atomic structure of $Ni_3In_2S_2$. **b**, STM images showing the topography before (left) and after (right) vacancy creation, demonstrating the remove of S atom at surface. **c**, STM images showing the topography before (left) and after (right) vacancy repair, demonstrating the filling of S atom. STM scanning parameters for (**b**-**c**): V_s=-300 mV; I_t=500 pA.

Supplementary Figure 18.

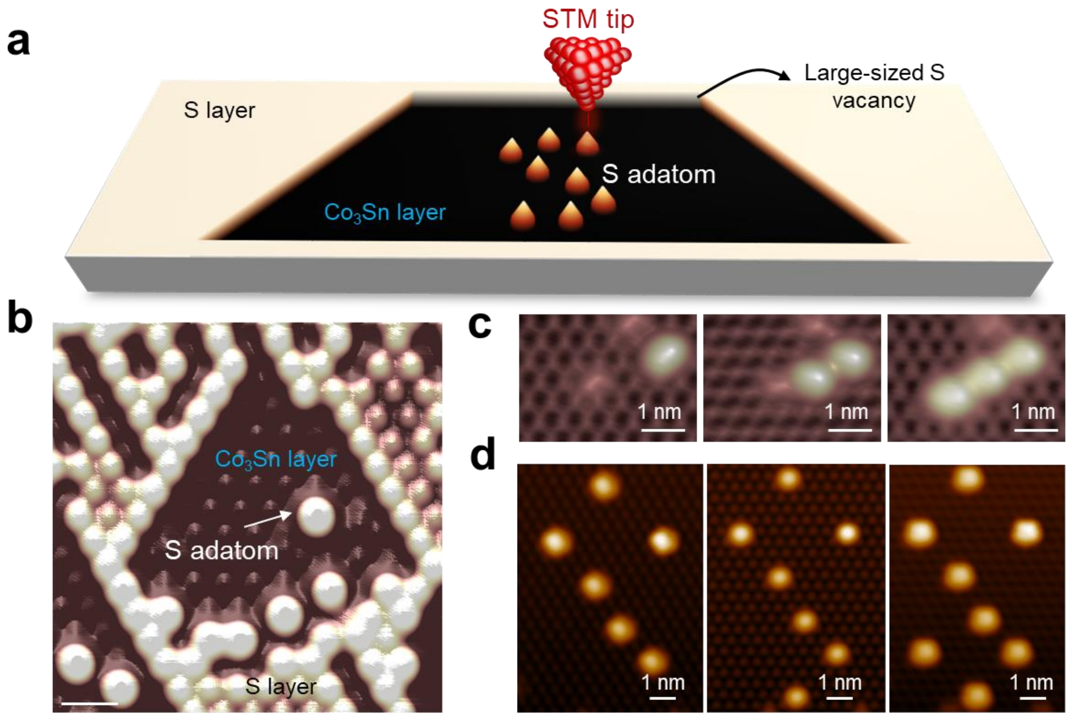

Supplementary Figure 18. Engineering of the S atoms inside large-size vacancy. a, Schematics of tip-assistant atomically precise engineering of S atoms inside large-size S vacancy. **b**, STM image showing that exposure of Co_3Sn layer when the S vacancies at S-terminated surface become relatively large. Some single S adatoms highlighted by the white arrows are observed. **c,** STM images showing the construction of S atomic chains with gradually increased length. **d**, Series of STM images showing the process to write a “S” letter by engineering the atomic S adatoms. The scale bar in all figures are 1 nm. $V_s = -400$ mV, $I_t = 500$ pA.

Supplementary Figure 19.

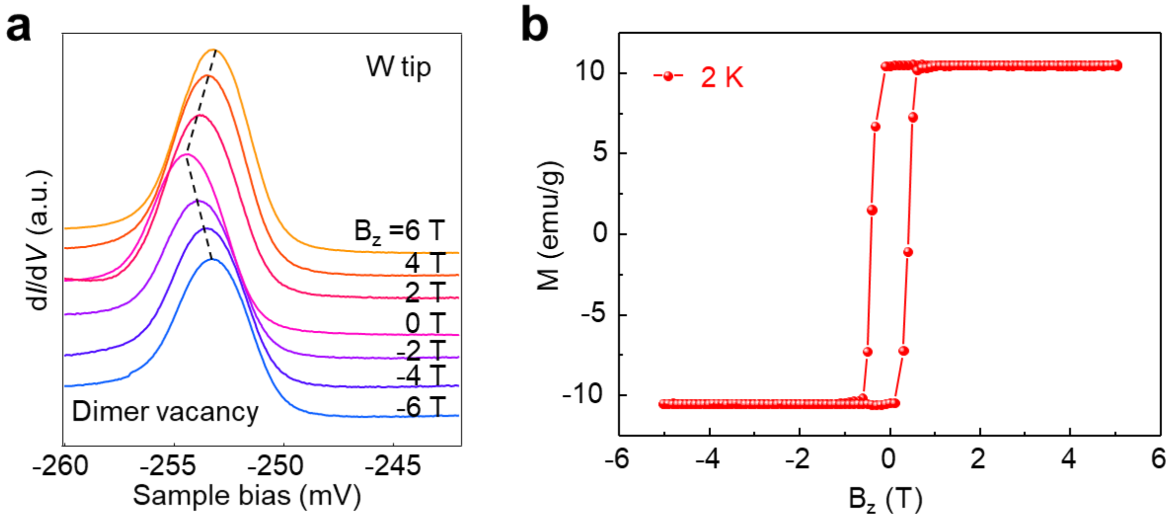

Supplementary Figure 19. Comparison with magnetic field d*I*/d*V* spectra obtained at S vacancies of $Co_3Sn_2S_2$ surface (a) and magnetization of bulk $Co_3Sn_2S_2$ crystal (b). The coercive field is approximately 4500 Oe, and the saturation magnetization is 10.38 emu/g. The magnetic moment corresponding to a cobalt atom is about 0.3 μ_B.
